# Crystal structure of (2*S*/2*R*,3*S*/3*R*)-3-hydroxy-2-phenyl­chroman-4-one

**DOI:** 10.1107/S2056989015001346

**Published:** 2015-01-28

**Authors:** Roumaissa Belguedj, Sofiane Bouacida, Hocine Merazig, Aissa Chibani, Abdelmalek Bouraiou

**Affiliations:** a Unité de Recherche de Chimie de l’Environnement et Moléculaire Structurale, CHEMS, Université Constantine1, 25000 , Algeria; b Département Sciences de la Matière, Faculté des Sciences Exactes et Sciences de la Nature et de la Vie, Université Oum El Bouaghi, Algeria

**Keywords:** crystal structure, flavone derivative, hydrogen bonds, C—H⋯π inter­actions

## Abstract

In the title mol­ecule, C_15_H_12_O_3_, the C atoms bearing the hy­droxy group and the phenyl ring are disordered over two sets of sites with refined occupancies of 0.573 (7) and 0.427 (7). There is also disorder of the phenyl ring but the hy­droxy group was refined as ordered. The dihedral angles between the benzene ring of the chromane ring system and the phenyl ring are 89.7 (2)° for the major component of disorder and 72.1 (3)° for the minor component. Both disorder components of the the di­hydro­pyran ring are in a half-chair conformation. In the crystal, mol­ecules are linked by pairs of O—H⋯O hydrogen bonds, forming inversion dimers with an *R*_2_^2^(10) graph-set motif. Weak C—H⋯π inter­actions link these dimers into ladders along [001].

## Related literature   

For the synthesis and applications of flavone derivatives, see: Gaspar *et al.* (2014 ▸); Huang *et al.* (2007 ▸); Yu *et al.* (2003 ▸); Phosrithong *et al.* (2012 ▸); Harborne & Williams (2000 ▸); Tanaka & Sugino (2001 ▸); Saxena *et al.* (1985 ▸). For the synthesis of the title compound, see: Juvale *et al.* (2013 ▸). For a related structure, see: Piaskowska *et al.* (2013 ▸).
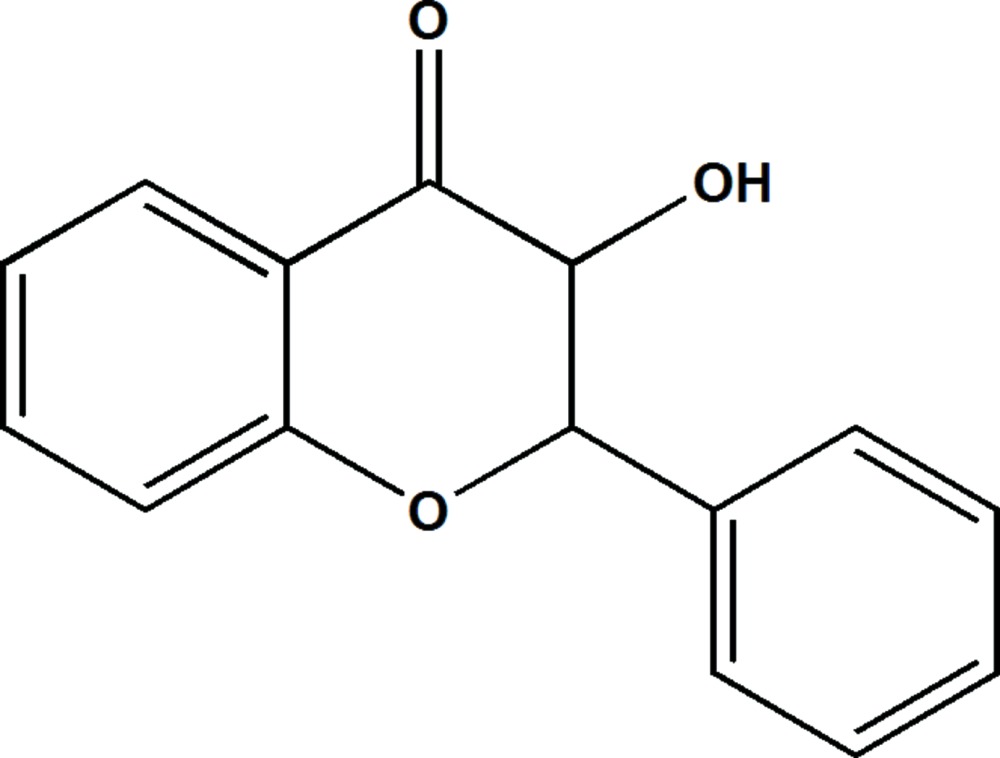


## Experimental   

### Crystal data  

C_15_H_12_O_3_
*M* *_r_* = 240.25
Monoclinic, 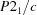

*a* = 5.3068 (3) Å
*b* = 26.7110 (18) Å
*c* = 9.4679 (6) Å
β = 117.431 (3)°
*V* = 1191.18 (13) Å^3^
*Z* = 4
Mo *K*α radiation
μ = 0.09 mm^−1^
*T* = 295 K
0.16 × 0.11 × 0.08 mm

### Data collection  

Bruker APEXII diffractometer
Absorption correction: multi-scan (*SADABS*; Sheldrick, 2002 ▸) *T*_min_ = 0.615, *T*_max_ = 0.745
6701 measured reflections
2356 independent reflections
1517 reflections with *I* > 2σ(*I*)
*R*_int_ = 0.031

### Refinement  

*R*[*F* ^2^ > 2σ(*F* ^2^)] = 0.058
*wR*(*F* ^2^) = 0.148
*S* = 1.06
2356 reflections
216 parameters
30 restraints
H atoms treated by a mixture of independent and constrained refinement
Δρ_max_ = 0.14 e Å^−3^
Δρ_min_ = −0.16 e Å^−3^

### 

Data collection: *APEX2* (Bruker, 2011 ▸); cell refinement: *SAINT* (Bruker, 2011 ▸); data reduction: *SAINT*; program(s) used to solve structure: *SIR2002* (Burla *et al.*, 2005 ▸); program(s) used to refine structure: *SHELXL97* (Sheldrick, 2015 ▸); molecular graphics: *ORTEP-3 for Windows* (Farrugia, 2012 ▸); software used to prepare material for publication: *WinGX* (Farrugia, 2012 ▸).

## Supplementary Material

Crystal structure: contains datablock(s) I. DOI: 10.1107/S2056989015001346/lh5747sup1.cif

Structure factors: contains datablock(s) I. DOI: 10.1107/S2056989015001346/lh5747Isup2.hkl

Supporting information file. DOI: 10.1107/S2056989015001346/lh5747Isup3.cml

**Fig. 1.**
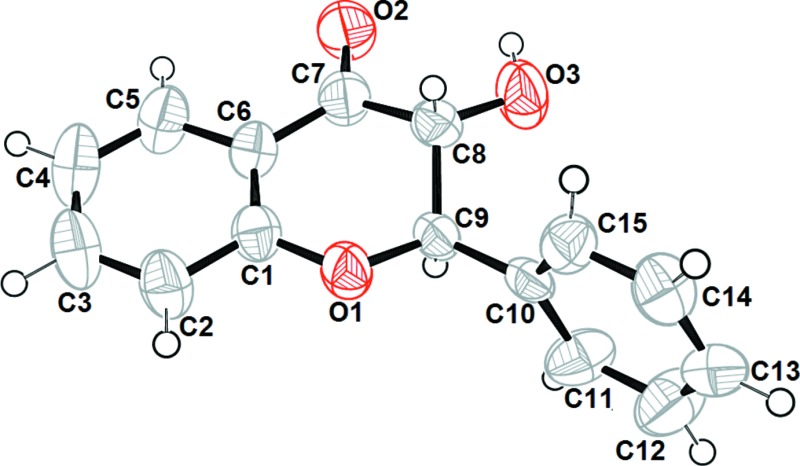
The molecule structure of the title compound. Displacement are drawn at the 30% probability level. H atoms are represented as small spheres of arbitrary radius. The minor component of disorder is not shown.

. DOI: 10.1107/S2056989015001346/lh5747fig1.tif
The mol­ecule structure of the title compound. Displacement are drawn at the 30% probability level. H atoms are represented as small spheres of arbitrary radius. The minor component of disorder is not shown.

**Fig. 2.**
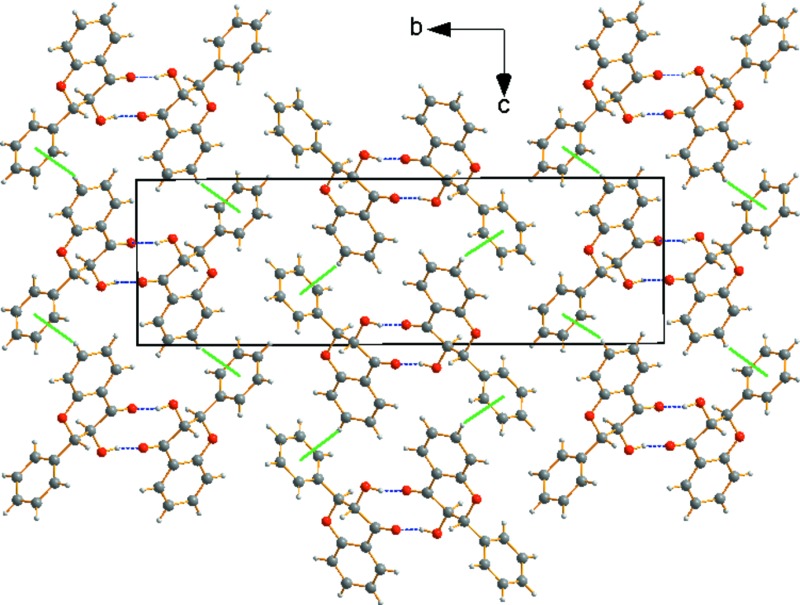
Part of the crystal structure of the title compound with hydrogen bonds shown as dashed lines and C—H···π intectations as green unbroken lines. The minor component of disorder is not shown.

. DOI: 10.1107/S2056989015001346/lh5747fig2.tif
Part of the crystal structure of the title compound with hydrogen bonds shown as dashed lines and C—H⋯π intectations as green unbroken lines. The minor component of disorder is not shown.

CCDC reference: 1044756

Additional supporting information:  crystallographic information; 3D view; checkCIF report

## Figures and Tables

**Table 1:** Hydrogen-bond geometry (, ) *Cg*1 and *Cg*2 are the centroids of the C10C15 and C10*A*C15*A* rings, respectively.

*D*H*A*	*D*H	H*A*	*D* *A*	*D*H*A*
O3H3*O*O2^i^	0.89(4)	2.04(4)	2.856(3)	153(4)
C3H3*A* *Cg*1^ii^	0.93	2.74	3.596(5)	153
C3H3*A* *Cg*2^ii^	0.93	2.92	3.756(5)	151

Symmetry codes: (i) 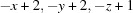; (ii) 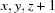.

## supplementary crystallographic information

### S1. Comment

Flavonoids are natural products derived from secondary metabolism of plants and play an important role in various biological processes (Harborne & Williams, 2000). All classes of flavonoids exhibit a variety of biological activities (Gaspar *et al.*, 2014; Huang *et al.*, 2007; Yu *et al.*, 2003; Phosrithong *et al.*, 2012). On the other hand, the Algar, Flynn and Oyamada (AFO) oxidation of substituted 2'-hydroxychalcones with alkaline hydrogen peroxide give flavonol derivatives (Juvale *et al.*, 2013). Dihydroflavonol was also obtained by this reaction (Saxena *et al.*, 1985; Tanaka & Sugino (2001). In this paper, we report the structure determination of the title compound resulting from the oxidation of 2'-hydroxychalcone using AFO reaction conditions.

The molecular structure of the title compound is shown in Fig. 1. The carbon atoms [C8 and C9] bearing the hydroxy group and the phenyl ring are disordered over two sets of sites with refined occupancies 0.573 (7) and 0.427 (7). This causes disorder of the phenyl ring [C10–C15] but the hydroxy group was refined as ordered. Atom O3 and the attached hydrogen atom occupy a single site. The dihedral angles between the benzene ring of the chromane ring system [C1–C6] and the phenyl ring are 89.7 (2)° for the major component of disorder [C10–C15] and 72.1 (3) for the minor component of disorder [C10A–C15A]. Both disorder components of the the dihydropyran are ring in a half-chair conformation. This type of geometry is comparable a published structure with a similar type of disorder (Piaskowska *et al.*, 2013).

In the crystal, pairs of molecules are linked by O—H···O hydrogen bonds (Table 1), forming inversion dimers with *R*_2_^2^(10) graph set motif. Weak C—H···pi interactions link these dimers into ladders along [001] (Fig. 2).

### S2. Experimental

The title compound was obtained by subjecting the (*E*)-1-(2-hydroxyphenyl)-3-phenylprop-2-en-1-one to Algar-Flynn-Oymanda (AFO) conditions using aqueous hydrogen peroxide in the presence of sodium hydroxide. Colorless crystals of the title compound I with melting point: 449–251 K (yield: 52%) were grown by slow evaporation of a solution of the title compound in diethylether. The 1H NMR spectra is in full agreement with the proposed structure (Tanaka & Sugino, 2001). The relative position of the hydroxyl and phenyl ring on the new heterocyclic ring could not be determined efficiently by NMR spectroscopy (J H2—H3 ≈ 12.4 Hz). However, the X-ray structure determination revealed a *trans*-configuration.

### S3. Refinement

Hydrogen atoms were located in differnce Fourier maps but introduced in calculated positions and treated as riding on their parent atom (C) with C—H = 0.93 and 0.98 Å and *U*_iso_(H) = 1.2*U*_eq_(C). The hydrogen atom of the hydroxy group was located in a difference map and refined isotropically with an O—H distance restraint of 0.85 (2) Å. The DELU and SADI commands in *SHELXL* (Sheldrick, 2008) were used in the refinment the disorder.

### Crystal data

**Table:**

C_15_H_12_O_3_	*F*(000) = 504
*M**_r_* = 240.25	*D*_x_ = 1.34 Mg m^−^^3^
Monoclinic, *P*2_1_/*c*	Mo *K*α radiation, λ = 0.71073 Å
Hall symbol: -P 2ybc	Cell parameters from 1712 reflections
*a* = 5.3068 (3) Å	θ = 2.5–23.2°
*b* = 26.7110 (18) Å	µ = 0.09 mm^−^^1^
*c* = 9.4679 (6) Å	*T* = 295 K
β = 117.431 (3)°	Prism, colorless
*V* = 1191.18 (13) Å^3^	0.16 × 0.11 × 0.08 mm
*Z* = 4	

### Data collection

**Table:**

Bruker APEXII diffractometer	2356 independent reflections
Radiation source: Enraf Nonius FR590	1517 reflections with *I* > 2σ(*I*)
Graphite monochromator	*R*_int_ = 0.031
CCD rotation images, thick slices scans	θ_max_ = 26.1°, θ_min_ = 2.9°
Absorption correction: multi-scan (*SADABS*; Sheldrick, 2002)	*h* = −5→6
*T*_min_ = 0.615, *T*_max_ = 0.745	*k* = −32→31
6701 measured reflections	*l* = −11→11

### Refinement

**Table:**

Refinement on *F*^2^	Primary atom site location: structure-invariant direct methods
Least-squares matrix: full	Secondary atom site location: difference Fourier map
*R*[*F*^2^ > 2σ(*F*^2^)] = 0.058	Hydrogen site location: inferred from neighbouring sites
*wR*(*F*^2^) = 0.148	H atoms treated by a mixture of independent and constrained refinement
*S* = 1.06	*w* = 1/[σ^2^(*F*_o_^2^) + (0.0475*P*)^2^ + 0.5327*P*] where *P* = (*F*_o_^2^ + 2*F*_c_^2^)/3
2356 reflections	(Δ/σ)_max_ < 0.001
216 parameters	Δρ_max_ = 0.14 e Å^−^^3^
30 restraints	Δρ_min_ = −0.16 e Å^−^^3^

### Special details

**Table:**

Geometry. All e.s.d.'s (except the e.s.d. in the dihedral angle between two l.s. planes) are estimated using the full covariance matrix. The cell e.s.d.'s are taken into account individually in the estimation of e.s.d.'s in distances, angles and torsion angles; correlations between e.s.d.'s in cell parameters are only used when they are defined by crystal symmetry. An approximate (isotropic) treatment of cell e.s.d.'s is used for estimating e.s.d.'s involving l.s. planes.
Refinement. Refinement of *F*^2^ against ALL reflections. The weighted *R*-factor *wR* and goodness of fit *S* are based on *F*^2^, conventional *R*-factors *R* are based on *F*, with *F* set to zero for negative *F*^2^. The threshold expression of *F*^2^ > σ(*F*^2^) is used only for calculating *R*-factors(gt) *etc*. and is not relevant to the choice of reflections for refinement. *R*-factors based on *F*^2^ are statistically about twice as large as those based on *F*, and *R*- factors based on ALL data will be even larger.

### Fractional atomic coordinates and isotropic or equivalent isotropic displacement parameters (Å^2^)

**Table:**

	*x*	*y*	*z*	*U*_iso_*/*U*_eq_	Occ. (<1)
O1	0.4498 (4)	0.85751 (6)	0.56442 (18)	0.0538 (5)	
O2	0.8643 (5)	0.98972 (7)	0.6185 (2)	0.0768 (6)	
O3	0.7507 (5)	0.93216 (8)	0.3576 (2)	0.0811 (7)	
C1	0.5095 (5)	0.88656 (9)	0.6949 (3)	0.0487 (6)	
C2	0.4263 (6)	0.86841 (11)	0.8046 (3)	0.0654 (8)	
H2A	0.3327	0.8379	0.7875	0.079*	
C3	0.4833 (7)	0.89589 (12)	0.9380 (3)	0.0782 (9)	
H3A	0.428	0.8837	1.0116	0.094*	
C4	0.6224 (8)	0.94163 (13)	0.9653 (3)	0.0826 (10)	
H4A	0.6623	0.9598	1.057	0.099*	
C5	0.7001 (7)	0.95974 (11)	0.8560 (3)	0.0694 (8)	
H5A	0.7888	0.9908	0.8725	0.083*	
C6	0.6481 (5)	0.93229 (9)	0.7201 (3)	0.0504 (6)	
C7	0.7425 (6)	0.95026 (10)	0.6059 (3)	0.0590 (7)	
C8	0.7356 (10)	0.91069 (15)	0.4871 (5)	0.0480 (15)	0.573 (7)
H8A	0.8973	0.888	0.5412	0.058*	0.573 (7)
C9	0.4642 (10)	0.88120 (18)	0.4324 (4)	0.0440 (14)	0.573 (7)
H9A	0.3063	0.905	0.386	0.053*	0.573 (7)
C8A	0.6023 (12)	0.9234 (2)	0.4446 (5)	0.048 (2)	0.427 (7)
H8AA	0.4052	0.9346	0.3836	0.058*	0.427 (7)
C9A	0.6092 (14)	0.8684 (2)	0.4818 (8)	0.0491 (18)	0.427 (7)
H9AA	0.8071	0.859	0.5506	0.059*	0.427 (7)
C10	0.4143 (17)	0.8417 (2)	0.3093 (6)	0.0438 (19)	0.573 (7)
C11	0.1560 (16)	0.8427 (3)	0.1717 (8)	0.066 (2)	0.573 (7)
H11A	0.0207	0.8667	0.1596	0.08*	0.573 (7)
C12	0.0997 (14)	0.8077 (3)	0.0521 (6)	0.082 (3)	0.573 (7)
H12A	−0.0731	0.8083	−0.0399	0.099*	0.573 (7)
C13	0.3018 (18)	0.7718 (3)	0.0702 (8)	0.075 (4)	0.573 (7)
H13A	0.2642	0.7484	−0.0098	0.09*	0.573 (7)
C14	0.5602 (16)	0.7708 (3)	0.2078 (9)	0.0589 (19)	0.573 (7)
H14A	0.6954	0.7468	0.2199	0.071*	0.573 (7)
C15	0.6165 (13)	0.8058 (3)	0.3273 (7)	0.0531 (19)	0.573 (7)
H15A	0.7893	0.8052	0.4194	0.064*	0.573 (7)
C10A	0.499 (2)	0.8360 (4)	0.3363 (9)	0.054 (3)	0.427 (7)
C11A	0.212 (2)	0.8324 (3)	0.2302 (10)	0.053 (2)	0.427 (7)
H11B	0.0805	0.8519	0.2447	0.063*	0.427 (7)
C12A	0.1204 (18)	0.7995 (4)	0.1023 (10)	0.063 (3)	0.427 (7)
H12B	−0.0718	0.797	0.0313	0.076*	0.427 (7)
C13A	0.317 (3)	0.7703 (3)	0.0806 (11)	0.063 (5)	0.427 (7)
H13B	0.2555	0.7482	−0.005	0.076*	0.427 (7)
C14A	0.604 (2)	0.7739 (5)	0.1867 (13)	0.081 (4)	0.427 (7)
H14B	0.735	0.7544	0.1721	0.097*	0.427 (7)
C15A	0.6950 (16)	0.8068 (5)	0.3145 (11)	0.066 (3)	0.427 (7)
H15B	0.8872	0.8093	0.3855	0.079*	0.427 (7)
H3O	0.843 (9)	0.9610 (11)	0.382 (5)	0.160 (18)*	

### Atomic displacement parameters (Å^2^)

**Table:**

	*U*^11^	*U*^22^	*U*^33^	*U*^12^	*U*^13^	*U*^23^
O1	0.0702 (11)	0.0484 (10)	0.0507 (10)	−0.0118 (8)	0.0346 (9)	−0.0009 (7)
O2	0.0990 (16)	0.0637 (12)	0.0829 (14)	−0.0364 (11)	0.0548 (13)	−0.0229 (10)
O3	0.1237 (19)	0.0708 (14)	0.0775 (13)	−0.0378 (13)	0.0709 (14)	−0.0188 (11)
C1	0.0544 (14)	0.0501 (14)	0.0424 (12)	0.0046 (12)	0.0229 (11)	0.0071 (11)
C2	0.085 (2)	0.0643 (17)	0.0572 (16)	0.0026 (15)	0.0411 (16)	0.0131 (13)
C3	0.110 (3)	0.082 (2)	0.0583 (18)	0.0144 (19)	0.0524 (18)	0.0184 (16)
C4	0.124 (3)	0.080 (2)	0.0492 (16)	0.016 (2)	0.0449 (19)	0.0012 (15)
C5	0.092 (2)	0.0647 (18)	0.0500 (15)	−0.0016 (15)	0.0312 (15)	−0.0051 (13)
C6	0.0578 (15)	0.0511 (15)	0.0414 (12)	0.0033 (12)	0.0220 (12)	0.0015 (11)
C7	0.0714 (17)	0.0544 (16)	0.0567 (16)	−0.0152 (14)	0.0340 (14)	−0.0079 (12)
C8	0.046 (3)	0.052 (3)	0.049 (3)	−0.006 (2)	0.024 (2)	0.003 (2)
C9	0.049 (3)	0.045 (3)	0.046 (3)	−0.008 (2)	0.029 (2)	−0.003 (2)
C8A	0.067 (5)	0.040 (4)	0.044 (4)	0.003 (3)	0.030 (4)	0.005 (3)
C9A	0.040 (4)	0.053 (4)	0.053 (4)	−0.001 (3)	0.021 (3)	−0.001 (3)
C10	0.046 (5)	0.046 (4)	0.053 (4)	−0.013 (3)	0.034 (4)	−0.005 (3)
C11	0.050 (3)	0.058 (5)	0.073 (5)	0.002 (3)	0.013 (4)	−0.007 (4)
C12	0.067 (5)	0.085 (6)	0.075 (5)	0.000 (4)	0.016 (4)	−0.021 (4)
C13	0.062 (6)	0.088 (9)	0.080 (8)	−0.031 (5)	0.038 (6)	−0.027 (6)
C14	0.068 (4)	0.044 (4)	0.073 (4)	0.017 (3)	0.040 (4)	0.002 (3)
C15	0.051 (4)	0.061 (4)	0.044 (3)	0.001 (4)	0.018 (3)	0.000 (3)
C10A	0.053 (7)	0.051 (6)	0.061 (5)	−0.012 (4)	0.029 (4)	−0.005 (4)
C11A	0.052 (6)	0.045 (5)	0.067 (7)	0.014 (4)	0.032 (6)	0.002 (4)
C12A	0.052 (5)	0.063 (5)	0.058 (5)	−0.013 (4)	0.011 (4)	−0.014 (5)
C13A	0.101 (11)	0.022 (6)	0.064 (9)	0.011 (6)	0.036 (8)	−0.008 (5)
C14A	0.090 (7)	0.078 (8)	0.084 (8)	−0.001 (6)	0.050 (6)	−0.012 (6)
C15A	0.059 (5)	0.078 (6)	0.076 (5)	−0.002 (5)	0.045 (5)	−0.004 (4)

### Geometric parameters (Å, º)

**Table:**

O1—C1	1.367 (3)	C8A—H8AA	0.98
O1—C9A	1.422 (4)	C9A—C10A	1.498 (4)
O1—C9	1.434 (4)	C9A—H9AA	0.98
O2—C7	1.213 (3)	C10—C11	1.39
O3—C8	1.389 (4)	C10—C15	1.39
O3—C8A	1.396 (4)	C11—C12	1.39
O3—H3O	0.885 (19)	C11—H11A	0.93
C1—C6	1.388 (3)	C12—C13	1.39
C1—C2	1.390 (3)	C12—H12A	0.93
C2—C3	1.369 (4)	C13—C14	1.39
C2—H2A	0.93	C13—H13A	0.93
C3—C4	1.389 (4)	C14—C15	1.39
C3—H3A	0.93	C14—H14A	0.93
C4—C5	1.367 (4)	C15—H15A	0.93
C4—H4A	0.93	C10A—C11A	1.39
C5—C6	1.394 (3)	C10A—C15A	1.39
C5—H5A	0.93	C11A—C12A	1.39
C6—C7	1.466 (3)	C11A—H11B	0.93
C7—C8	1.532 (4)	C12A—C13A	1.39
C7—C8A	1.534 (5)	C12A—H12B	0.93
C8—C9	1.509 (4)	C13A—C14A	1.39
C8—H8A	0.98	C13A—H13B	0.93
C9—C10	1.502 (4)	C14A—C15A	1.39
C9—H9A	0.98	C14A—H14B	0.93
C8A—C9A	1.507 (5)	C15A—H15B	0.93
			
C1—O1—C9A	115.6 (3)	O3—C8A—H8AA	109.9
C1—O1—C9	117.0 (2)	C9A—C8A—H8AA	109.9
C9A—O1—C9	31.3 (2)	C7—C8A—H8AA	109.9
C8—O3—C8A	29.9 (3)	O1—C9A—C10A	107.9 (5)
C8—O3—H3O	113 (3)	O1—C9A—C8A	111.8 (4)
C8A—O3—H3O	113 (3)	C10A—C9A—C8A	113.0 (6)
O1—C1—C6	122.6 (2)	O1—C9A—H9AA	108
O1—C1—C2	117.1 (2)	C10A—C9A—H9AA	108
C6—C1—C2	120.3 (2)	C8A—C9A—H9AA	108
C3—C2—C1	119.4 (3)	C11—C10—C15	120
C3—C2—H2A	120.3	C11—C10—C9	117.4 (5)
C1—C2—H2A	120.3	C15—C10—C9	122.6 (5)
C2—C3—C4	121.0 (3)	C12—C11—C10	120
C2—C3—H3A	119.5	C12—C11—H11A	120
C4—C3—H3A	119.5	C10—C11—H11A	120
C5—C4—C3	119.4 (3)	C11—C12—C13	120
C5—C4—H4A	120.3	C11—C12—H12A	120
C3—C4—H4A	120.3	C13—C12—H12A	120
C4—C5—C6	120.8 (3)	C14—C13—C12	120
C4—C5—H5A	119.6	C14—C13—H13A	120
C6—C5—H5A	119.6	C12—C13—H13A	120
C1—C6—C5	119.0 (2)	C13—C14—C15	120
C1—C6—C7	119.7 (2)	C13—C14—H14A	120
C5—C6—C7	121.3 (2)	C15—C14—H14A	120
O2—C7—C6	124.0 (2)	C14—C15—C10	120
O2—C7—C8	120.2 (2)	C14—C15—H15A	120
C6—C7—C8	114.5 (2)	C10—C15—H15A	120
O2—C7—C8A	119.7 (3)	C11A—C10A—C15A	120
C6—C7—C8A	114.0 (3)	C11A—C10A—C9A	122.6 (8)
C8—C7—C8A	27.1 (2)	C15A—C10A—C9A	117.3 (8)
O3—C8—C9	110.3 (3)	C12A—C11A—C10A	120
O3—C8—C7	111.8 (3)	C12A—C11A—H11B	120
C9—C8—C7	107.9 (3)	C10A—C11A—H11B	120
O3—C8—H8A	108.9	C11A—C12A—C13A	120
C9—C8—H8A	108.9	C11A—C12A—H12B	120
C7—C8—H8A	108.9	C13A—C12A—H12B	120
O1—C9—C10	107.8 (4)	C12A—C13A—C14A	120
O1—C9—C8	110.9 (3)	C12A—C13A—H13B	120
C10—C9—C8	115.6 (5)	C14A—C13A—H13B	120
O1—C9—H9A	107.4	C15A—C14A—C13A	120
C10—C9—H9A	107.4	C15A—C14A—H14B	120
C8—C9—H9A	107.4	C13A—C14A—H14B	120
O3—C8A—C9A	109.9 (4)	C14A—C15A—C10A	120
O3—C8A—C7	111.2 (3)	C14A—C15A—H15B	120
C9A—C8A—C7	105.9 (4)	C10A—C15A—H15B	120
			
C9A—O1—C1—C6	18.5 (4)	O2—C7—C8A—O3	32.0 (6)
C9—O1—C1—C6	−16.6 (4)	C6—C7—C8A—O3	−164.2 (3)
C9A—O1—C1—C2	−161.2 (4)	C8—C7—C8A—O3	−67.0 (5)
C9—O1—C1—C2	163.7 (3)	O2—C7—C8A—C9A	151.4 (4)
O1—C1—C2—C3	179.4 (2)	C6—C7—C8A—C9A	−44.8 (5)
C6—C1—C2—C3	−0.3 (4)	C8—C7—C8A—C9A	52.5 (5)
C1—C2—C3—C4	0.1 (5)	C1—O1—C9A—C10A	−175.4 (5)
C2—C3—C4—C5	0.9 (5)	C9—O1—C9A—C10A	−75.0 (9)
C3—C4—C5—C6	−1.7 (5)	C1—O1—C9A—C8A	−50.5 (6)
O1—C1—C6—C5	179.8 (2)	C9—O1—C9A—C8A	49.9 (5)
C2—C1—C6—C5	−0.5 (4)	O3—C8A—C9A—O1	−177.4 (4)
O1—C1—C6—C7	−1.6 (4)	C7—C8A—C9A—O1	62.3 (6)
C2—C1—C6—C7	178.1 (2)	O3—C8A—C9A—C10A	−55.4 (7)
C4—C5—C6—C1	1.5 (4)	C7—C8A—C9A—C10A	−175.7 (5)
C4—C5—C6—C7	−177.1 (3)	O1—C9—C10—C11	107.8 (5)
C1—C6—C7—O2	179.8 (3)	C8—C9—C10—C11	−127.5 (4)
C5—C6—C7—O2	−1.6 (4)	O1—C9—C10—C15	−73.4 (6)
C1—C6—C7—C8	−13.0 (4)	C8—C9—C10—C15	51.3 (6)
C5—C6—C7—C8	165.6 (3)	C15—C10—C11—C12	0
C1—C6—C7—C8A	16.8 (4)	C9—C10—C11—C12	178.8 (6)
C5—C6—C7—C8A	−164.6 (3)	C10—C11—C12—C13	0
C8A—O3—C8—C9	51.2 (5)	C11—C12—C13—C14	0
C8A—O3—C8—C7	−68.9 (4)	C12—C13—C14—C15	0
O2—C7—C8—O3	−28.7 (5)	C13—C14—C15—C10	0
C6—C7—C8—O3	163.6 (3)	C11—C10—C15—C14	0
C8A—C7—C8—O3	68.3 (4)	C9—C10—C15—C14	−178.7 (6)
O2—C7—C8—C9	−150.2 (3)	O1—C9A—C10A—C11A	50.0 (9)
C6—C7—C8—C9	42.1 (4)	C8A—C9A—C10A—C11A	−74.2 (8)
C8A—C7—C8—C9	−53.2 (5)	O1—C9A—C10A—C15A	−126.3 (6)
C1—O1—C9—C10	175.6 (4)	C8A—C9A—C10A—C15A	109.5 (6)
C9A—O1—C9—C10	80.1 (8)	C15A—C10A—C11A—C12A	0
C1—O1—C9—C8	48.1 (5)	C9A—C10A—C11A—C12A	−176.2 (9)
C9A—O1—C9—C8	−47.4 (5)	C10A—C11A—C12A—C13A	0
O3—C8—C9—O1	178.2 (3)	C11A—C12A—C13A—C14A	0
C7—C8—C9—O1	−59.4 (5)	C12A—C13A—C14A—C15A	0
O3—C8—C9—C10	55.1 (5)	C13A—C14A—C15A—C10A	0
C7—C8—C9—C10	177.6 (4)	C11A—C10A—C15A—C14A	0
C8—O3—C8A—C9A	−48.9 (5)	C9A—C10A—C15A—C14A	176.4 (9)
C8—O3—C8A—C7	68.1 (5)		

### Hydrogen-bond geometry (Å, º)

Cg1 and Cg2 are the centroids of the C10–C15 and C10A–C15A rings, respectively.

**Table:**

*D*—H···*A*	*D*—H	H···*A*	*D*···*A*	*D*—H···*A*
O3—H3*O*···O2^i^	0.89 (4)	2.04 (4)	2.856 (3)	153 (4)
C3—H3*A*···*Cg*1^ii^	0.93	2.74	3.596 (5)	153
C3—H3*A*···*Cg*2^ii^	0.93	2.92	3.756 (5)	151

Symmetry codes: (i) −*x*+2, −*y*+2, −*z*+1; (ii) *x*, *y*, *z*+1.
