# Supplementary figures and images for: Crystal structure of (2S/2R,3S/3R)-3-hydroxy-2-phenyl­chroman-4-one
Source: Acta Crystallogr E Crystallogr Commun. 2015 Jan 28;71(Pt 2):o131–2. doi: 10.1107/S2056989015001346 (PMC4384603; doi:10.1107/S2056989015001346)

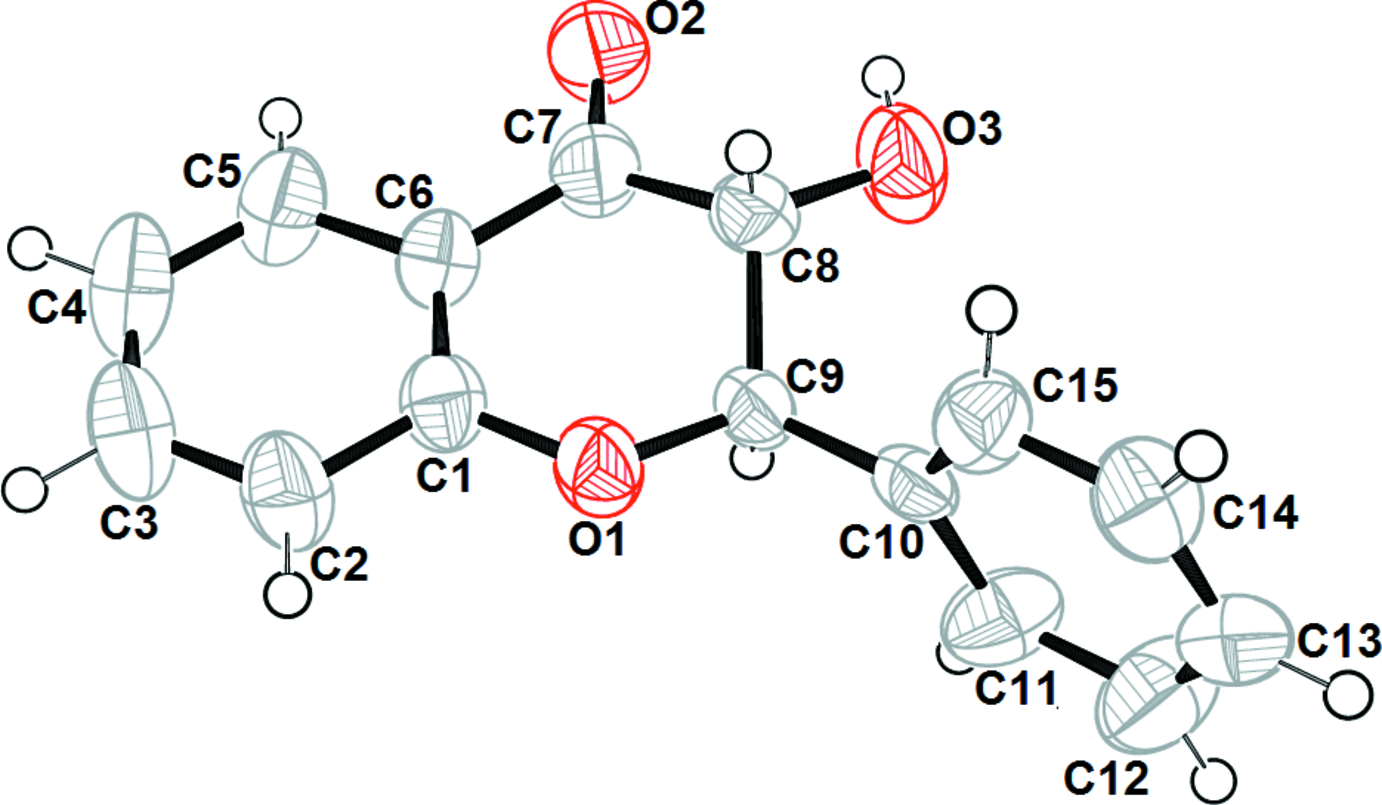

Supplement: Supplementary file 4 [file e-71-0o131-fig1.tif]

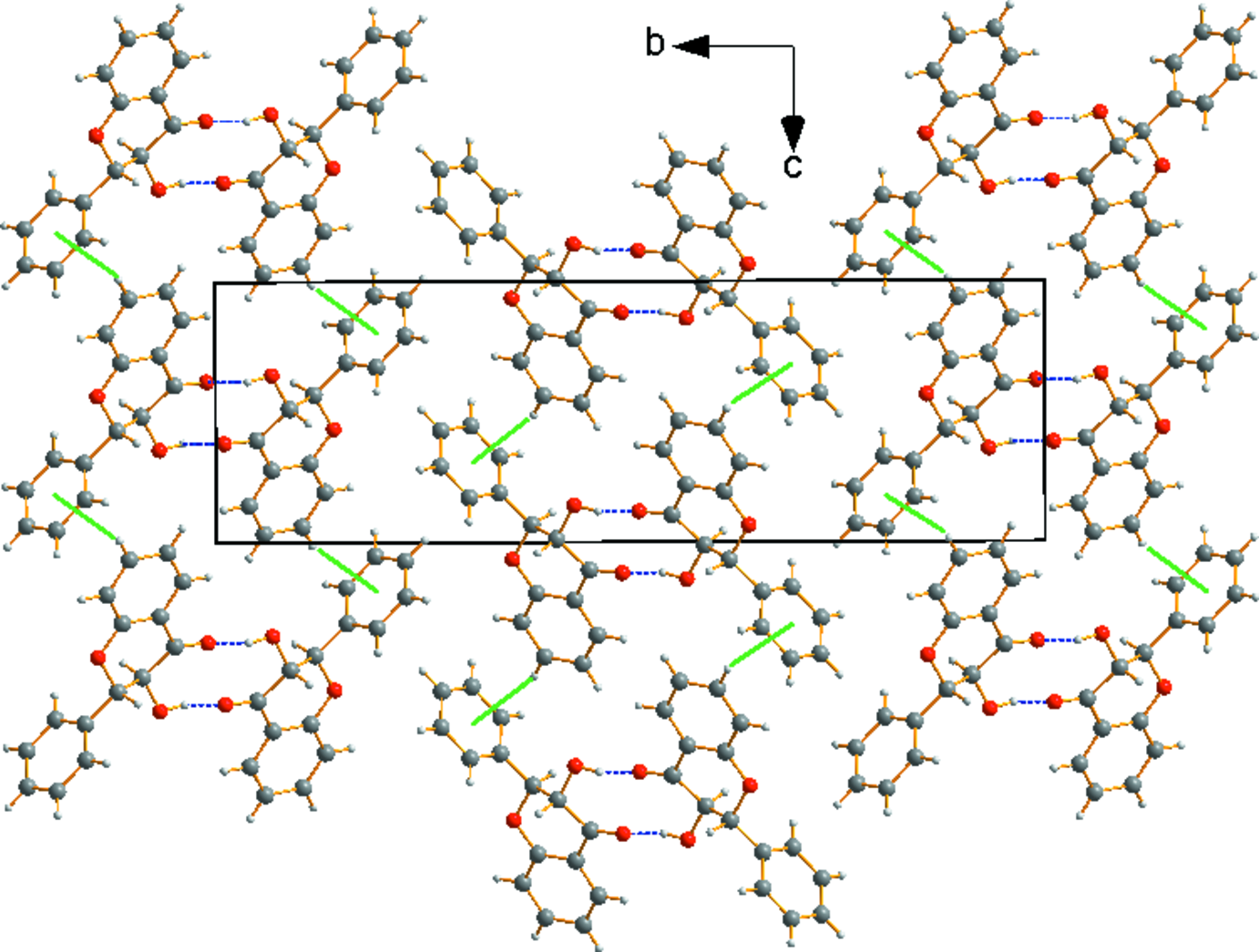

Supplement: Supplementary file 5 [file e-71-0o131-fig2.tif]
